# Eugenol Attenuates Cerebral Ischemia-Reperfusion Injury by Enhancing Autophagy *via* AMPK-mTOR-P70S6K Pathway

**DOI:** 10.3389/fphar.2020.00084

**Published:** 2020-02-21

**Authors:** Xiaowei Sun, Dongyan Wang, Tingting Zhang, Xuejian Lu, Fangfang Duan, Lili Ju, Xiaotong Zhuang, Xicheng Jiang

**Affiliations:** ^1^ Department of Acupuncture and Moxibustion, The First Affiliated Hospital, Heilongjiang University of Chinese Medicine, Harbin, China; ^2^ Department of Acupuncture and Moxibustion, The Second Affiliated Hospital, Heilongjiang University of Chinese Medicine, Harbin, China; ^3^ Department of Integrated Chinese and Western Medicine, The First Affiliated Hospital, Heilongjiang University of Chinese Medicine, Harbin, China; ^4^ Department of Chinese Medicine Clinical Foundation, School of Basic Medical Sciences, Heilongjiang University of Chinese Medicine, Harbin, China; ^5^ Department of Synopsis of the Golden Chamber, School of Basic Medical Sciences, Heilongjiang University of Chinese Medicine, Harbin, China

**Keywords:** eugenol, cerebral I/R injury, autophagy, HT 22, AMPK/mTOR/P70S6K pathway

## Abstract

Eugenol, as an active compound isolated from Acorus gramineus, has been shown to protect against cerebral ischemia-reperfusion (I/R) injury. Nonetheless, the detailed neuroprotective mechanisms of eugenol in cerebral I/R injury have not been elaborated. In the present study, cerebral I/R injury model was established by middle cerebral artery occlusion (MCAO) in rats. HT22 cells were subjected to oxygen-glucose deprivation/reperfusion (OGD/R) to mimic cerebral I/R injury *in vitro*. The results showed that eugenol pre-treatment relieved cerebral I/R injury as evidenced by improving neurological deficits and reducing infarct volume. Autophagy was induced by MCAO, which was further promoted by eugenol administration. Moreover, rapamycin, an activator of autophagy, promoted eugenol-induced decreases in neurological score, infarct volume, brain water content, and apoptosis. However, pretreatment with 3-MA, an inhibitor of autophagy, led to the opposite results. Similarly, eugenol pretreatment increased the viability and restrained apoptosis of OGD/R-challenged HT22 cells. OGD/R-induced autophagy was strengthened by eugenol. Mechanically, eugenol promoted autophagy through regulating AMPK/mTOR/P70S6K signaling pathway *in vivo* and *in vitro*. In conclusion, pretreatment with eugenol attenuated cerebral I/R injury by inducing autophagy *via* AMPK/mTOR/P70S6K signaling pathway.

## Introduction

Stroke, caused by cerebral artery occlusion and subsequent hypoperfusion, is one of the most serious diseases threatening human's health and lives. About 800,000 patients are diagnosed with stroke in the United States each year (Roger et al., 2012). With an ageing population, the financial burden caused by stroke is increasing worldwide (Forouzanfar et al., 2015; Feigin et al., 2016). For people over 60, stroke is the most common cause of permanent disabilities and the second leading cause of mortality and dementia (Chamorro et al., 2016). Recombinant plasminogen activator is the only FDA-approved thrombolytic agent for treating stroke at present (Lekoubou et al., 2017), but the narrow treatment time windows and side effects may limit its widespread application. Therefore, it is necessary to develop new drugs and methods for treating stroke.

Autophagy, as a basic biological process that degrades and recycles damaged organelles and intracellular macromolecules, participates in multiple pathological and physiological processes. It has been documented that autophagy takes part in the progression of various diseases, such as neurodegenerative disorders (Kumar et al., 2018), diabetes (Barlow and Thomas, 2015), obesity (Fu et al., 2019), and so on. Notably, growing evidence has indicated that autophagy is also involved in many ischemic diseases, including myocardial infarction (Aisa et al., 2017), renal ischemia reperfusion injury (Hou et al., 2019), and cerebral ischemia reperfusion injury (Zhang et al., 2013), etc. Ischemic stroke may result in the induction of autophagy, but its role in stroke is still controversial. A series of researches suggest that autophagy functions as a double-edged sword that presents both beneficial and harmful effects on cerebral neurons in response to ischemia stimulus (Wang et al., 2018).

Eugenol is one of active compounds of a well-known traditional Chinese medicine Acorus gramineus, which has attracted wide attention for its antiinflammatory (El-Kady et al., 2019), antiapoptotic (Ekinci Akdemir et al., 2019), antioxidant (Ekinci Akdemir et al., 2019), and antitumor (Fangjun and Zhijia, 2018) effects. A previous study showed that eugenol could protect against hepatic ischemia-reperfusion injury (Abd El Motteleb et al., 2014). Importantly, studies have suggested that eugenol has beneficial effects on cerebral ischemic injury (Won et al., 1998; Ahmad et al., 2018). In addition, a similar compound methyleugenol was demonstrated to alleviate cerebral ischemic injury *via* inhibiting oxidative stress, inflammation, and apoptosis (Choi et al., 2010). However, it is not clear whether eugenol attenuates cerebral ischemia-reperfusion injury through regulating autophagy, which needs to be elucidated.

In the present study, we investigated whether eugenol could protect against ischemic stroke *via* regulating autophagy in a rat model of cerebral ischemia-reperfusion injury *in vivo* and oxygen glucose deprivation-reperfusion (OGD/R)-challenged mouse neuronal HT22 cells *in vitro*.

## Materials and Methods

### Experimental Animals

Eight-to-ten-week-old male Sprague-Dawley rats (250–300 g) were purchased from ChangSheng biotechnology co., Ltd. (Liaoning, China). The animal experiments were performed in accordance with the Guidelines for the Care and Use of Laboratory Animals and approved by the ethics committee of Center for Drug Evaluation, Heilongjiang University of Chinese Medicine.

### Animal Model and Drug Administration

#### Experiment I

The rats were randomly divided into sham, ischemia/reperfusion (I/R), I/R+eugenol 50 mg/kg, and I/R+eugenol 100 mg/kg groups (n = 18 per group). Middle cerebral artery occlusion (MCAO) was carried out to induce cerebral I/R injury. Briefly, after anesthesia by intraperitoneal injection of 40 mg/kg pentobarbital sodium, the rats were fixed in the supine position. The right common carotid artery (CCA), external carotid artery (ECA), and internal carotid artery (ICA) were isolated and exposed. Then, the CCA and ECA were ligated proximally. A nylon suture with a diameter of 0.285 mm was advanced from a small puncture on CCA up to the ICA for about 18 mm to occlude the middle cerebral artery. After 120 min, the nylon suture was slowly withdrawn to restore blood flow. The sham rats were subjected to the same surgery without MCAO. The rats in eugenol groups were administrated with 50 mg/kg or 100 mg/kg eugenol (Aladdin Biological Co., Ltd, Shanghai, China) once a day for 15 days by gavage before MCAO. The other rats were administrated with equal volume vehicle by gavage.

#### Experiment II

To evaluate the role of autophagy in the neuroprotection of eugenol against cerebral I/R injury, the rats were randomly divided into I/R, I/R+ eugenol 100 mg/kg, I/R+ eugenol 100 mg/kg+ rapamycin, and I/R+ eugenol 100 mg/kg+3-Methyladenine (3-MA) groups (n = 18 per group). After pretreatment with eugenol 100 mg/kg for 15 days, rapamycin (30 ng in 10 μl normal saline, Meilunbio, Dalian, China), 3-MA (30 μg in 10 μl normal saline, Aladdin, Shanghai, China), or normal saline (10 μl, vehicle control) was injected into the right cerebral ventricle of rats at 10 min before MCAO, respectively.

### Neurobehavioral Evaluation

At 24 h after the reperfusion, neurological deficit in rats was determined by a blinded investigator using the modified neurological severity scoring (mNSS) system as previously described (Zhao et al., 2019). The mNSS scores range from 0 to 18, with higher score indicating serious neurological deficit.

### Quantification of Infarct Volume

The brain tissues were collected at 24 h after reperfusion and then the 2,3,5-triphenyltetrazolium chloride (TTC) staining was performed to assess infarct volume. In brief, the brain tissues were cut into five 2-mm slices and then incubated in 0.4% TTC solution (Sangon Biotech, Shanghai, China) for 15 min at 37°C. The images were taken by a digital camera and the infarct volume was quantitatively analyzed by Image-ProPlus Analysis Software (Media Cybernetics, Inc., Bethesda, MD, USA). The infarct volume was calculated as follow: Infarct volume (%) = (∑Infarct area × thickness)/(∑whole brain area × thickness) × 100%.

### Brain Water Content

At 72 h after the reperfusion, the brains of rats were collected after euthanasia and immediately weighed as the wet weight. Then the brains were put in an oven (100°C) for 72 h and weighed as the dry weight. The brain water content was calculated using the following formula: (wet weight–dry weight)/wet weight × 100%.

### Western Blotting Analysis

The following primary antibodies were adopted for Western blotting: Beclin-1 (1:1,000, Proteintech, Wuhan, China), LC3I/II (1:1,000, Proteintech), p62 (1:2,000, Proteintech), mTOR (1:1,000, Cell Signaling Technology, Trask Lane Danvers, MA, USA), p-mTOR (1:1,000, Cell Signaling Technology), AMPKα (1:1,000, Cell Signaling Technology), p-AMPKα (1:1,000, Cell Signaling Technology), P70S6K (1:1,000, Cell Signaling Technology), p-P70S6K (1:1,000, Cell Signaling Technology), and β-actin (1:1,000, Santa Cruz, Finnell Street Dallas, TX, USA). HRP-labeled Goat Anti-Rabbit IgG (1:5000, Beyotime, Haimen, China) was used as the secondary antibody. The right cerebral tissues of ipsilateral hemisphere or HT22 cells were lysed in Cell lysis buffer for Western and IP (Beyotime) containing 1 mM PMSF (Beyotime) and phosphatase inhibitors for 5 min on ice. Then the quantified protein samples were separated on sodium dodecyl sulfate polyacrylamide gel electrophoresis and blotted onto polyvinylidene fluoride membranes (Millipore, Billerica, MA, USA). Blocking was performed at room temperature for 1 h using 5% skimmed milk. Subsequently, the membranes were incubated with the above primary antibodies at 4°C overnight, respectively. After incubation with the secondary antibody at 37°C for 45 min, the immunoreactive bands were detected using BeyoECL Plus (Beyotime). The bands were quantified using Gel-Pro-Analyzer software (Media Cybernetics). The optical density of the target protein was normalized on β-actin, and then the fold change relative to sham, I/R, or OGD/R group was calculated and shown.

### Immunofluorescence Staining

At 24 h after the reperfusion, the brain tissues from ipsilateral hemisphere were collected and fixed in 4% paraformaldehyde. After embedding in paraffin and cutting into 5-μm sections, the antigen was retrieved in citrate buffer for 10 min using a microwave oven. Then the sections were blocked in goat serum (Solarbio, Beijing, China) for 15 min and incubated with LC3 primary antibody (1:200, Proteintech) at 4°C overnight. Cy3-labeled Goat Anti-Rabbit IgG (1:200, Beyotime) was added to the sections for 1 h at room temperature. DAPI solution (Biosharp, Hefei, China) was added for nuclear counterstaining. The staining was visualized under a fluorescence microscope (Olympus, Tokyo, Japan) at a magnification of 400×. The integrated intensity/field was quantified using Image Pro-Plus software (Media Cybernetics) in one slice from each rat.

### TUNEL Assay

The 5-μm brain sections were obtained as described above. The apoptosis of brain tissues in the ischemic penumbra area was assessed using an In Situ Cell Death Detection Kit (Roche, Basel, Switzerland) according to the manufacturer’ protocol. The images were acquired using an inverted microscope (Olympus) at a magnification of 400×.

### Cell Culture and Treatments

HT22 cells were obtained from Zhong Qiao Xin Zhou Biotechnology Co., Ltd (Shanghai, China) and cultured in Dulbecco’s Modified Eagle’s Medium (DMEM, Hyclone, Logan, UT, USA) supplemented with 10% fetal bovine serum (FBS, Biological Industries, Kibbutz Beit Haemek, Israel) at 37 °C in 5% CO_2_.

To mimic cerebral I/R injury *in vitro*, HT22 cells were subjected to oxygen-glucose deprivation/reperfusion (OGD/R) treatment. Briefly, HT22 cells were incubated in DMEM without glucose and FBS at 37 °C in 0.5% O_2_ and 5% CO_2_ for 12 h and then cultured in normal culture condition as described above for 24 h. To determine the protective effect of eugenol, HT22 cells were pretreated with various concentrations of eugenol at 24 h before the exposure to OGD/R. The inhibitor of AMPK pathway, compound C (10 μM, MedChemExpress, Shanghai, China) was added to HT22 cells at 1 h before the administration with 100 μM eugenol.

### Cell Viability Measurement

The viability of HT22 cells receiving various treatments was measured with MTT assay. In brief, HT22 cells in 96-well plates were added with MTT solution (0.5 mg/ml, Sigma, Saint Louis, MO, USA) and incubated at 37°C for 4 h. Then the purple crystals were dissolved in 150 μl DMSO for 10 min in the dark. The optical density at 570 nm was obtained on a microplate reader (BioTek, Winooski, VT, USA).

### AnnexinV/PI Staining for Apoptosis Detection

Annexin V-FITC Apoptosis Detection Kit (Beyotime) was used to detect apoptosis of HT22 cells. Briefly, HT22 cells subjected to various treatments were harvested and resuspended in 195 μl Annexin V-FITC binding buffer. Then, HT22 cells were added with 5 μl AnnexinV-FITC and 10 μl PI solution, followed by incubation for 15 min away from light at room temperature. The apoptosis of HT22 cells was detected on a flow cytometer (ACEA Biosciences, San Diego, CA, USA).

### Statistical Analysis

All data are presented as mean ± standard deviation and were analyzed using GraphPad Prism 8.0 software (La Jolla, CA, USA). One-way analysis of variance, followed by Tukey post hoc test was carried out to evaluate statistical differences among the experimental groups. For neurological score, differences were compared using Kruskal-Wallis test followed by Dunn’s test. *P*-value less than 0.05 was considered as statistical significance.

## Results

### Eugenol Treatment Attenuated Cerebral I/R Injury in MCAO Rats

**Figure 1:**
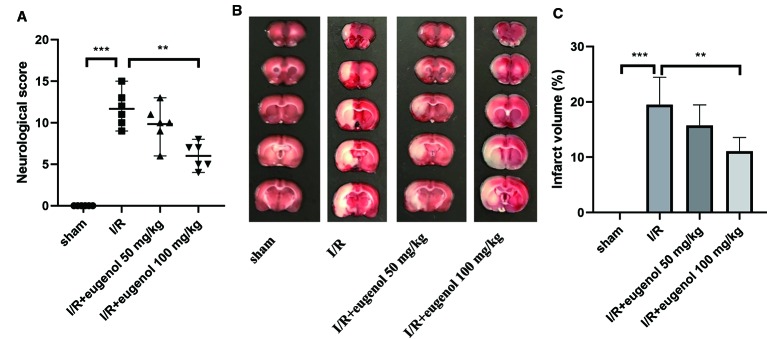
Eugenol treatment attenuated cerebral I/R injury in middle cerebral artery occlusion (MCAO) rats. **(A)** The neurological deficits were detected by mNSS scoring at 24 h after reperfusion. **(B)** Triphenyltetrazolium chloride (TTC) staining assay was performed to evaluate infarct volume. **(C)** The infarct volume of different groups was calculated and shown. Each experimental datum was presented as mean±standard deviation (n = 6 animals per group). **P < 0.01, ***P < 0.001 versus the specified group.

As shown in **Figure 1A**, the effect of eugenol on MCAO-induced neurological deficits was assessed by neurological score. The neurological score was significantly increased in I/R injury group compared with sham group, indicating a severe neurologic injury. However, eugenol treatment effectively attenuated the neurological deficit in MCAO rats. TTC staining results showed that the infarct volume was larger in I/R injury group than that in sham group, whereas administration with eugenol obviously reduced the infarct volume of rats challenged with MCAO (**Figures 1B, C**). Thus, treatment with eugenol relieved cerebral I/R injury in rats.

### Eugenol Enhanced Autophagy in the Brains of MCAO Rats

**Figure 2:**
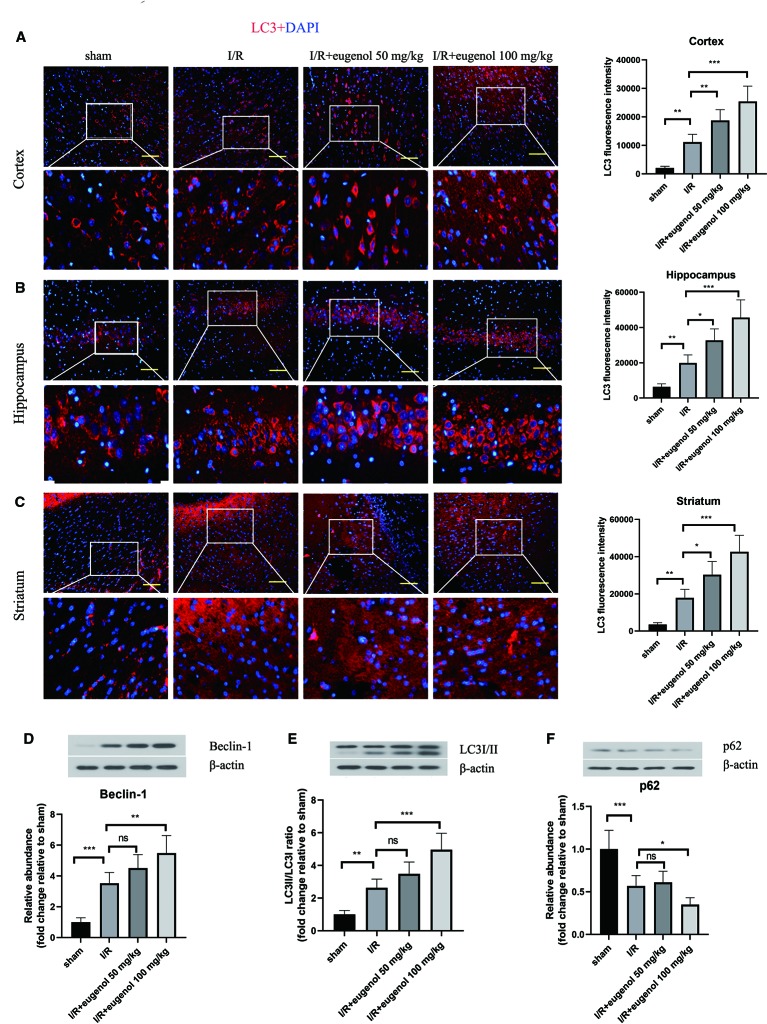
Eugenol enhanced autophagy in the brains of middle cerebral artery occlusion (MCAO) rats. The expression of LC3 in the cortical penumbra area **(A)**, CA1 hippocampus **(B)**, and striatum **(C)** was detected by immunofluorescent assay (Scale bar = 50 μm). The expression of Beclin-1 **(D)**, LC3I/II **(E)**, and p62 **(F)** in brain tissues was assessed by Western blotting. Each experimental datum was presented as mean±standard deviation (n = 6 animals per group). *P < 0.05, **P < 0.01, ***P < 0.001 versus the specified group. ns, no significant difference.

To assess the role of eugenol in the regulation of autophagy during cerebral I/R injury, the expression of LC3, an autophagy marker, was detected by immunofluorescence staining. As presented in **Figures 2A–C**, the expression of LC3 in cortical penumbra area, CA1 hippocampus, and striatum was increased in response to MCAO, which was further enhanced by eugenol treatment. In addition, MCAO challenge also upregulated Beclin-1 level and LC3II/LC3I ratio, while downregulated p62 level. Similarly, the increase in Beclin-1 level and LC3II/LC3I ratio and decrease in p62 level were further promoted by eugenol administration (**Figures 2D–F**), indicating that eugenol promoted autophagy during cerebral I/R injury in rats.

### Eugenol Alleviated Cerebral I/R Injury Through Regulating Autophagy

**Figure 3:**
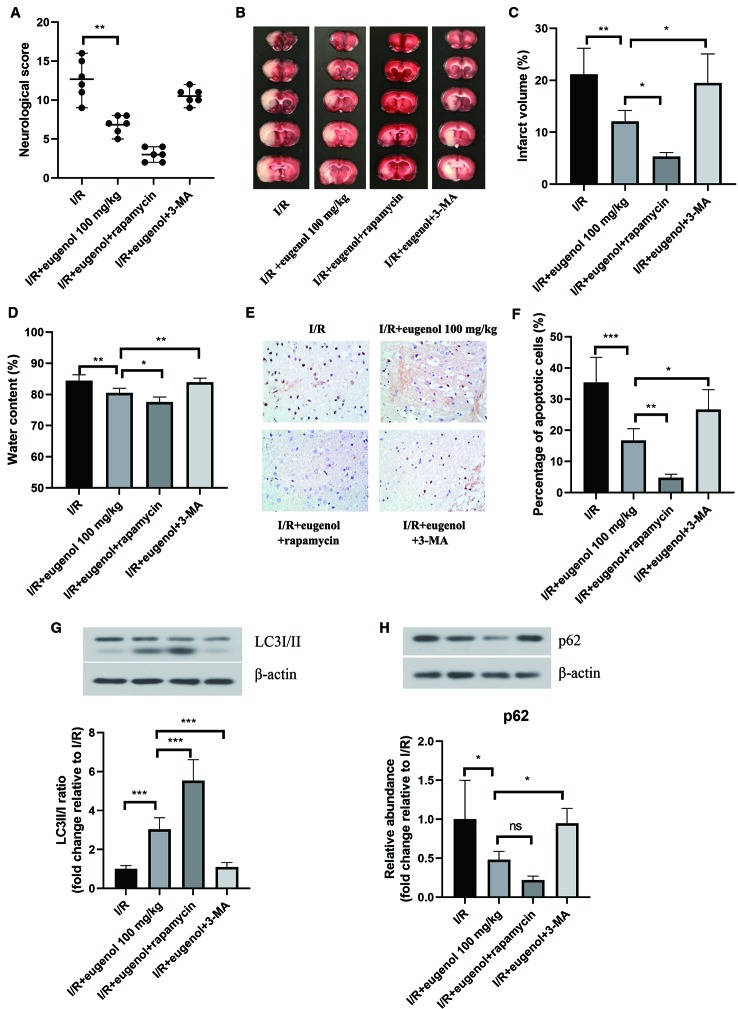
Eugenol alleviated cerebral ischemia-reperfusion (I/R) injury through regulating autophagy. **(A)** The neurological deficits in different treatment groups were assessed by neurological score at 24 h after reperfusion. **(B)** Triphenyltetrazolium chloride (TTC) staining assay was adopted to evaluate infarct volume. **(C)** The infarct volume of different groups was calculated and shown. **(D)** The water content in brain was detected and shown. **(E, F)** TUNEL assay was performed to determine the apoptosis in brain tissues. The expression of LC3I/II **(G)**, and p62 **(H)** in brain tissues was assessed by Western blotting. Each experimental datum was presented as mean±standard deviation (n = 6 animals per group). *P < 0.05, **P < 0.01, ***P < 0.001 versus the specified group. ns, no significant difference.

To investigate the role of autophagy in eugenol-mediated neuroprotection, the rats were treated with eugenol in combination with rapamycin (an autophagy activator) or 3-MA (an autophagy inhibitor). As shown in **Figure 3A**, eugenol-induced decrease in neurological score was enhanced by rapamycin, but partly reversed by 3-MA. Moreover, as assessed by TCC staining, combination with rapamycin further reduced the infarct volume, whereas combination with 3-MA showed an opposite result (**Figures 3B, C**). The water content in brain was decreased by eugenol administration, which was significantly enhanced by rapamycin, but restrained by 3-MA (**Figure 3D**). Moreover, the infarct volume of MCAO mice was reduced by rapamycin, while increased by 3-MA (**Supplementary Figure 1A, B**). Additionally, the apoptosis in brain tissues was evaluated by TUNEL (**Figure 3E**). The apoptotic cells in the ischemic penumbra area of brain tissues was remarkably lessened by eugenol. As expected, combination with rapamycin further suppressed apoptosis, while 3-MA treatment obviously promoted apoptosis (**Figure 3F**). The levels of cleaved caspase-3 and Bad were reduced, but Bcl-2 level was enhanced by eugenol administration (**Supplementary Figure 2**). As shown in **Figures 3G, H**, the upregulated LC3II/I ratio and downregulated p62 level were promoted by rapamycin, but counteracted by 3-MA. All these results indicated that autophagy induction was involved in the beneficial effect of eugenol against cerebral I/R injury.

### Eugenol Regulated AMPK/mTOR/P70S6K Pathway in Cerebral I/R Injury

**Figure 4:**
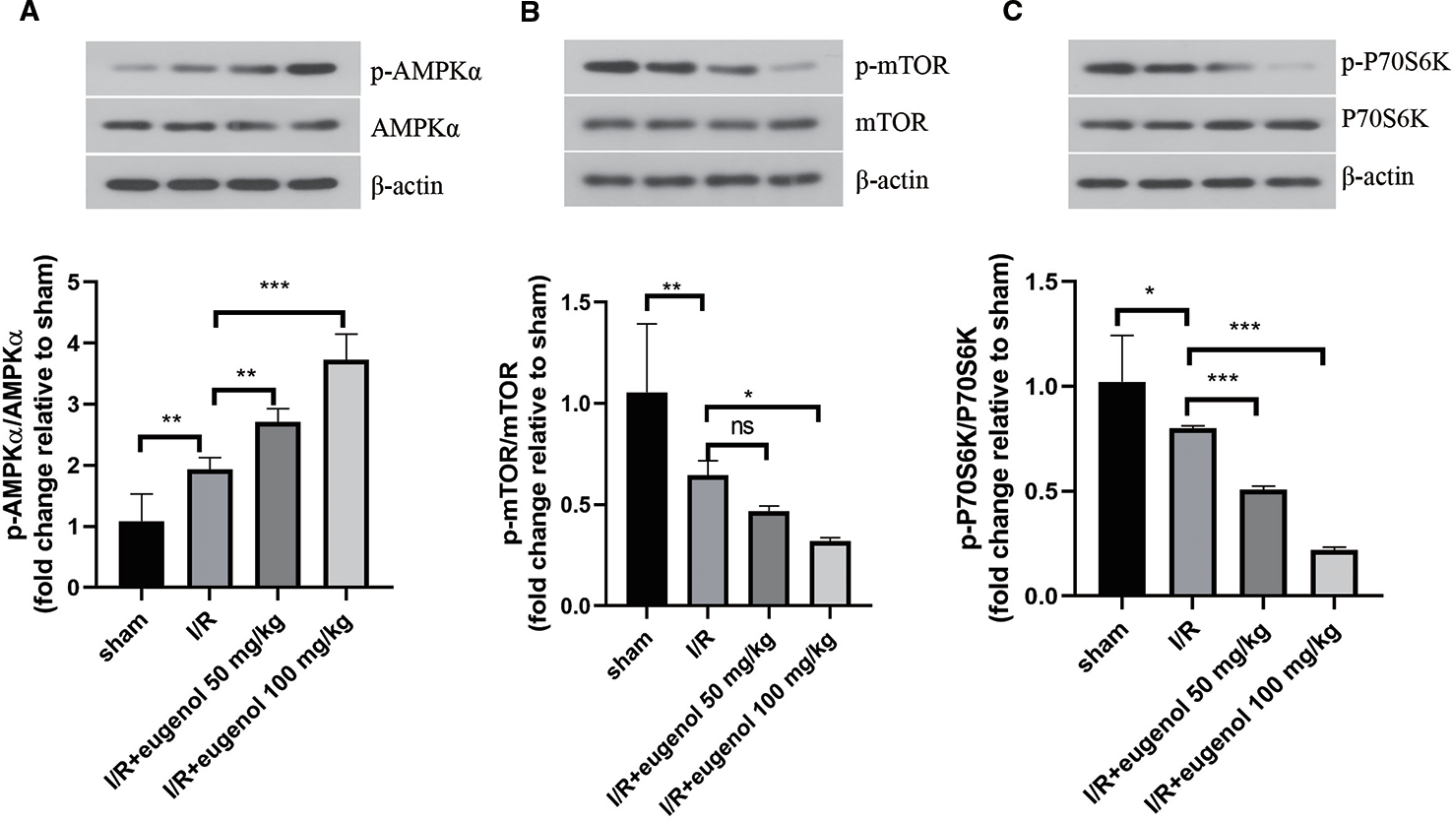
Eugenol regulated AMPK/mTOR/P70S6K pathway in cerebral I/R injury. **(A–C)** The protein levels of p-AMPK, AMPKα, p- mTOR, mTOR, p-P70S6K, and P70S6K in brain tissues were detected by Western blotting assay. Each experimental datum was presented as mean±standard deviation (n = 6 animals per group). *P < 0.05, **P < 0.01, ***P<0.001 versus the specified group. ns, no significant difference.

Next, the molecular mechanisms of eugenol in cerebral I/R injury was explored. As illustrated in **Figures 4A–C**, the p-AMPKα/AMPKα ratio was raised, while the p-mTOR/mTOR and p-P70S6K/P70S6K ratios were declined after challenge with MCAO. Treatment with eugenol could intensify the above changes dramatically. Therefore, AMPK/mTOR/P70S6K pathway participated in the protective mechanisms of eugenol in cerebral I/R injury.

### Eugenol Protected HT22 Cells Against OGD/R

**Figure 5:**
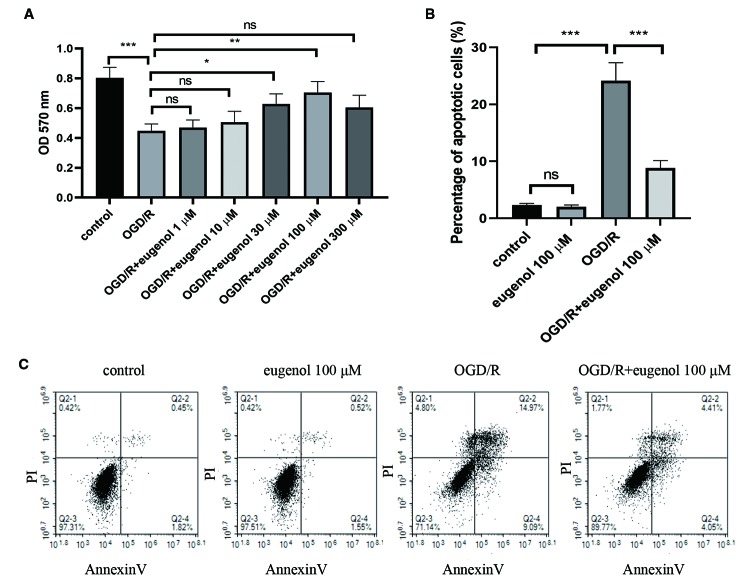
Eugenol protected HT22 cells against oxygen-glucose deprivation/reperfusion (OGD/R). **(A)** The viability of HT22 cells after exposure to OGD/R was detected by MTT assay. **(B, C)** The apoptosis of HT22 cells was determined by AnnexinV/PI staining and the percentage of apoptotic cells was calculated. Each experimental datum was presented as mean±standard deviation (n = 3; three independent experiments). *P < 0.05, **P < 0.01, ***P < 0.01 versus the specified group. ns, no significant difference.

As shown in **Figure 5A**, OGD/R led to a significant decrease in the viability of HT22 cells, which was mitigated by the incubation with 30 or 100 μM eugenol. Since 100 μM eugenol presented the optimal effect, we chose this dose in the following experiments. Moreover, eugenol treatment notably suppressed OGD/R-induced apoptosis in HT22 cells (**Figures 5B, C**). These findings suggested that eugenol attenuated OGD/R-induced damage in HT22 cells.

### Eugenol Improved Cell Viability of HT22 Cells Through Inducing Autophagy *via* AMPK/mTOR/P70S6K Pathway

**Figure 6:**
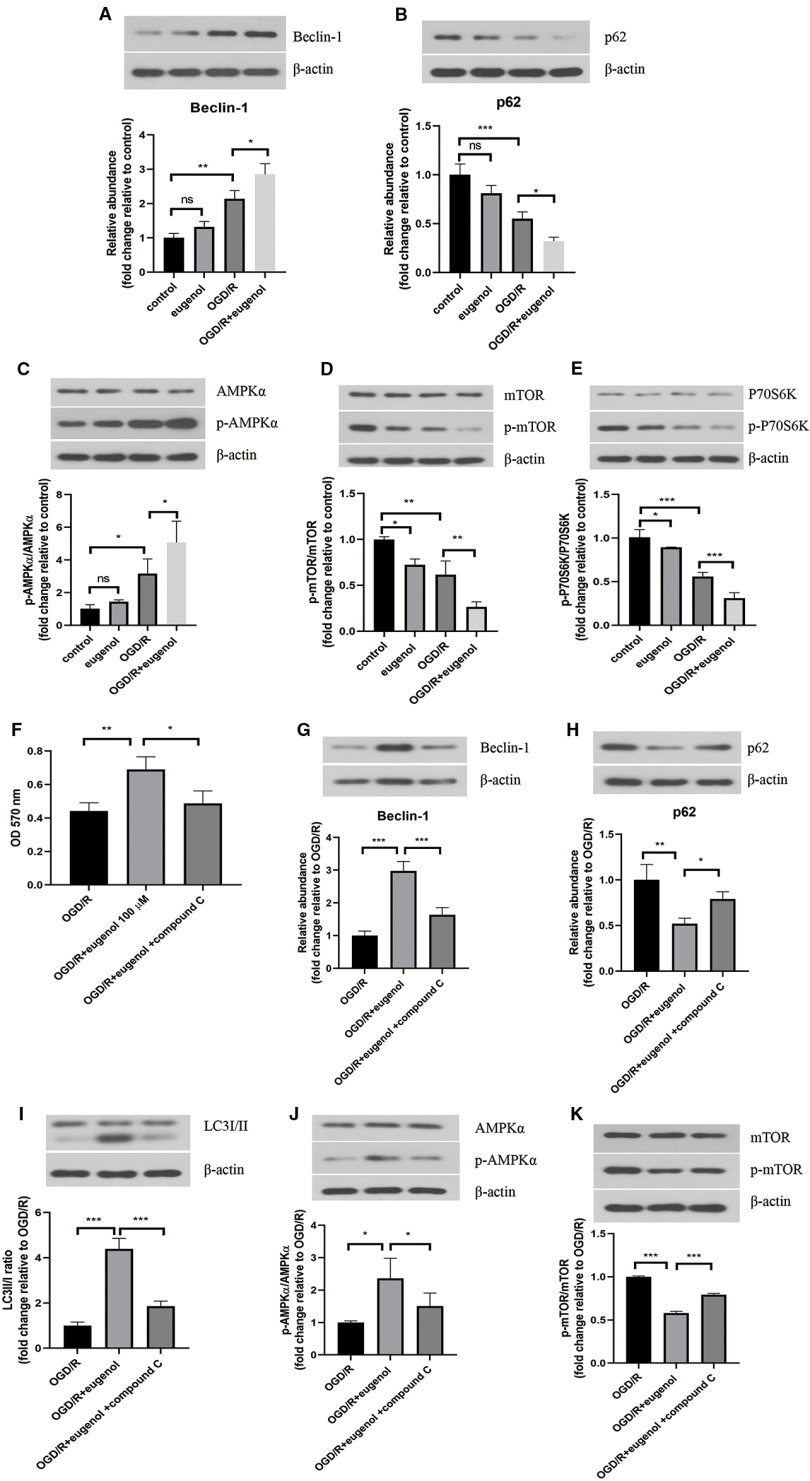
Eugenol improved cell viability of HT22 cells through inducing autophagy *via* AMPK/mTOR/P70S6K pathway. Western blotting was performed to assess Beclin-1 **(A)** and p62 **(B)** levels in HT22 cells. **(C–E)** The protein levels of p-AMPK, AMPKα, p- mTOR, mTOR, p-P70S6K, and P70S6K in HT22 cells were detected by Western blotting assay. **(F)** The viability of HT22 cells was detected by MTT assay. The protein levels of Beclin-1 **(G)**, p62 **(H)**, LC3I/II **(I)**, p-AMPK, AMPKα **(J)**, p-mTOR, and mTOR **(K)** in HT22 cells was detected by Western blotting assay. Each experimental datum was presented as mean±standard deviation (n = 3; three independent experiments). *P < 0.05, **P < 0.01, ***P < 0.01 versus the specified group. ns, no significant difference.

After exposure to OGD/R, an obvious increase in Beclin-1 level, but decrease in p62 level was found. As might have been expected, a higher Beclin-1 level and a lower p62 level was induced by eugenol as compared with OGD/R group (**Figures 6A, B**). Moreover, OGD/R-induced apoptosis in HT22 cells was attenuated by rapamycin, but intensified by 3-MA (**Supplementary Figure 1B**). To explore the signaling pathway through which eugenol regulated autophagy, the protein levels of p-AMPKα, AMPKα, p-mTOR, mTOR, p-P70S6K, and P70S6K were assessed by Western blotting. As presented in **Figures 6C–E**, eugenol treatment enhanced the p-AMPKα/AMPKα ratio, while reduced the p-mTOR/mTOR and p-P70S6K/P70S6K ratios. To further determine the involvement of AMPK/mTOR/P70S6K pathway in eugenol-mediated autophagy, an AMPK inhibitor compound C was added. As shown in **Figure 6F**, the increased viability of HT22 cells induced by eugenol was counteracted by compound C. More importantly, compound C restrained eugenol-induced autophagy by reducing Beclin-1 level, LC3II/I ratio, and p-AMPKα/AMPKα ratio, while increasing p62 level and p-mTOR/mTOR ratio (**Figures 6G–K**). Therefore, eugenol promoted the survival of HT22 cells *via* inducing AMPK/mTOR/P70S6K-dependent autophagy.

## Discussion

Stroke is the major cause of death and physical disability all around the world, accounting for half of hospitalized patients with acute neurological deficit (Lo et al., 2003). In the current study, we investigated the effect of eugenol on ischemic stroke in a rat MCAO model *in vivo* and OGD/R-induced HT22 cells *in vitro*. The results revealed that eugenol relieved cerebral I/R injury and concomitant neurological deficit. Moreover, induction of autophagy was involved in the protective mechanisms of eugenol against cerebral I/R injury. Mechanically, eugenol induced autophagy through regulating AMPK/mTOR/P70S6K signaling pathway.

Autophagy, as one hotspot of biomedical research, is a lysosome-mediated degradation process of cellular components. When the number of damaged organelles increases, outside pathogens invade, or abnormal accumulation of proteins occurs, the cell content is wrapped in vesicular membrane structure to form autophagosomes that are further integrated with lysosomes to form autolysosomes (Sica et al., 2015). Therefore, the cell content degrades to small molecular substances that prepare for aerobic respiration (Hansen and Johansen, 2011). In 1995, Nitatori et al firstly confirmed the occurrence of autophagy in neurocytes after cerebral ischemia using transmission electron microscopy (Nitatori et al., 1995). In recent years, mounting evidence indicates that cerebral I/R injury closely relates to autophagy (Zhang et al., 2019a). The research by Zhang et al. showed that astragaloside IV treatment exerted neuroprotective effects on cerebral I/R injury *via* the promotion of autophagy(Zhang et al., 2019b). LncRNA SNHG12-induced autophagy activation alleviated cerebral I/R injury, which was partially reversed by an autophagy inhibitor 3-MA(Yao et al., 2019). All these studies revealed that autophagy plays neuroprotective roles after cerebral I/R injury. Reports also demonstrate that autophagy is deleterious for ischemic brain (Zhou et al., 2017; Feng et al., 2018). This discrepancy might be caused by different animal strains, ischemic models, and time of ischemia. Indeed, the role of autophagy in cell death/survival remains debated and complicated. In addition, Wang et al suggested that whether autophagy is beneficial or harmful in ischemic stroke depended on the degree and duration of autophagy (Wang et al., 2018). In this study, we demonstrated that autophagy was beneficial for ischemic stroke and eugenol pretreatment-mediated autophagy activation could further reduce infarct volume and improve neurological deficit. Therefore, autophagy induction participated in the protective effect of eugenol against ischemic stroke.

Autophagy is an evolutionarily conserved process controlled by multiple autophagy-related proteins. Beclin-1 is the first discovered key factor that can make autophagy-related proteins localize in autophagosome membranes and regulate the formation and maturation of autophagosomes (Sun et al., 2008; Itakura and Mizushima, 2009). During autophagy, LC3 is transferred into LC3-I by ATG4-mediated dihydroxylation, followed by production of LC3-II in autophagosome, which is the biological marker for autophagosome formation (Schaaf et al., 2016). P62 binds to LC3, which contributes to the recruitment of cell content into autophagosomes for degradation (Mizushima and Komatsu, 2011). P62 is degraded during autophagy process (Kim et al., 2014). According to our results, eugenol treatment further contributed to MCAO or OGD/R-induced increase in Beclin-1 level and LC3II/I ratio, and decrease in p62 level. From these results, we suggested that autophagy induction was involved in the protective mechanisms of eugenol against cerebral I/R injury.

Autophagy and apoptosis have been shown to be interconnected with each other. It is well documented that apoptosis inhibition is involved in the cytoprotective effect of autophagy. Apoptosis is triggered if p62-mediated autophagy is prohibited (Goodall et al., 2016). Cheng et al. suggested that the neuronal apoptosis was inhibited by recombinant OPN *via* enhancing autophagy in subarachnoid hemorrhage-induced brain injury (Sun et al., 2019). Moreover, previous studies also proved that autophagy induction alleviated apoptosis in Parkinson’s disease (Lin et al., 2014) and stroke (Wu et al., 2018). Recent studies have indicated that autophagy exhibited a protective effect *via* suppressing the apoptosis cascade in ischemic stroke (Zhang et al., 2013; Sun et al., 2018b). Consistent with these observations, our results indicated that the inhibitory effect of eugenol against MCAO-induced apoptosis was promoted by rapamycin, while counteracted by 3-MA. Furthermore, the apoptosis in OGD/R-challenged HT22 cells was attenuated by eugenol. These findings revealed that eugenol protected against apoptosis after cerebral I/R injury through inducing autophagy.

Finally, the molecular mechanisms of eugenol in regulating autophagy were investigated. AMPK/mTOR has been recognized as a crucial pathway in the regulation of autophagy (Kim et al., 2011). AMPK, as a metabolism and energy regulator, is verified to be a promoter of autophagy (Meley et al., 2006). Specifically, AMPK enhances autophagy *via* the direct activation of ULK1 (Kim et al., 2011). AMPK-mediated the activation of autophagy represents a protective mechanism in ischemic stroke (Shen et al., 2017). mTOR, an evolutionarily conserved serine/threonine kinase, takes part in multiple biological processes, including cell proliferation, autophagy, protein synthesis, and metabolism (Weichhart, 2012). The activation of mTOR could inhibit autophagy *via* controlling ULK1 ubiquitylation (Nazio et al., 2013). In addition, suppressing the activation of P70S6K, a key downstream kinase of mTOR, can promote autophagy (Sun et al., 2018a). It has been shown that eugenol activates AMPK phosphorylation, but suppresses mTOR and P70S6K phosphorylation, which attenuates hepatic steatosis and fibrosis (Jo et al., 2014). In the present study, the ratio of p-AMPKα/AMPKα was increased, while p-mTOR/mTOR and p-P70S6K/P70S6K ratios were reduced by MCAO or OGD/R, which was further strengthened by eugenol. Additionally, compound C, an inhibitor of AMPK, reversed eugenol-induced autophagy and survival of HT22 cells after exposure to OGD/R. Therefore, the neuroprotective effects of eugenol were attributed to inducing AMPK/mTOR/P70S6K pathway-dependent autophagy.

There are some limitations in this article. Although we evaluated the preventive potential of eugenol on cerebral I/R injury, however, the therapeutic effects of eugenol after stroke and the time window for administration need to been investigated in the future. Secondly, it is noteworthy that the adult rats (8–10 weeks old) were used in our study, whereas ischemic stroke often occurs in the elderly. Hence, the efficacy of eugenol on stroke in aged rodents needs to be evaluated in future experiments.

## Conclusion

Taken together, the findings proved that the protective effect of eugenol against cerebral I/R injury was ascribed to autophagy induction *via* regulating AMPK/mTOR/P70S6K pathway. Our results provide some new insights into the protective mechanisms of eugenol against ischemic stroke.

## Data Availability Statement

The datasets generated for this study are available on request to the corresponding author.

## Ethics Statement

The animal experiments were performed in accordance with the Guidelines for the Care and Use of Laboratory Animals and approved by the ethics committee of Center for Drug Evaluation, Heilongjiang University of Chinese Medicine.

## Author Contributions

Study concepts and design: XS, DW, and XJ. Experimental studies: XS, DW, TZ, and XL. Reagents, materials, and analysis tools: FD, LJ, and XZ. Writing manuscript: XS and DW. Review and editing manuscript: XJ. Funding acquisition: XJ. Project administration: XJ.

## Funding

This study was supported by grants from the National Nature Science Foundation of China (81673865 and 81503669), the Outstanding Training Foundation of Heilongjiang University of Chinese Medicine (2019JC05) and the Outstanding Innovative Talents Support Plan of Heilongjiang University of Chinese Medicine (2018RCD11).

## Conflict of Interest

The authors declare that the research was conducted in the absence of any commercial or financial relationships that could be construed as a potential conflict of interest.

## Supplementary Material

Figure S1 Cerebral I/R injury was attenuated by autophagy. Cerebral I/R injury was induced by MCAO in mice. Autophagy was promoted by pretreatment with rapamycin, or inhibited by pretreatment with 3-MA. **(A)** After reperfusion for 24 h, TTC staining assay was performed to evaluate infarct volume. **(B)** The infarct volume of different groups was calculated and shown. HT22 cells were pretreated with rapamycin (1 μM) or 3-MA (5 mM) for 1 h, and then subjected to OGD/R. **(C)** The apoptosis of HT22 cells was determined by AnnexinV/PI staining and the percentage of apoptotic cells was calculated. Each experimental datum was presented as mean±standard deviation (n = 6 for A&B, n = 3 for C). *P < 0.05, **P < 0.01 versus the indicated group.

Figure S2 Effect of eugenol on the expression of apoptotic markers. **(A–C)** The protein levels of cleaved caspase-3, Bad, and Bcl-2 in the brain tissues were detected by Western blotting assay. Each experimental datum was presented as mean±standard deviation (n = 6). *P < 0.05, ***P < 0.001 versus the indicated group.

## References

[B1] Abd El Motteleb D. M. Selim S. A. Mohamed A. M. (2014). Differential effects of eugenol against hepatic inflammation and overall damage induced by ischemia/re-perfusion injury. J. Immunotoxicol 11 (3), 238–245. 10.3109/1547691X.2013.832444 24099633

[B2] Ahmad N. Ahmad R. Alam M. A. Ahmad F. J. (2018). Quantification and brain targeting of eugenol-loaded surface modified nanoparticles through intranasal route in the treatment of Cerebral Ischemia. Drug Res. (Stuttg) 68 (10), 584–595. 10.1055/a-0596-7288 29669380

[B3] Aisa Z. Liao G. C. Shen X. L. Chen J. Li L. Jiang S. B. (2017). Effect of autophagy on myocardial infarction and its mechanism. Eur. Rev. Med. Pharmacol. Sci. 21 (16), 3705–3713. 10.26355/eurrev_201708_13288 28925470

[B4] Barlow A. D. Thomas D. C. (2015). Autophagy in diabetes: beta-cell dysfunction, insulin resistance, and complications. DNA Cell Biol. 34 (4), 252–260. 10.1089/dna.2014.2755 25665094

[B5] Chamorro A. Dirnagl U. Urra X. Planas A. M. (2016). Neuroprotection in acute stroke: targeting excitotoxicity, oxidative and nitrosative stress, and inflammation. Lancet Neurol. 15 (8), 869–881. 10.1016/S1474-4422(16)00114-9 27180033

[B6] Choi Y. K. Cho G. S. Hwang S. Kim B. W. Lim J. H. Lee J. C. (2010). Methyleugenol reduces cerebral ischemic injury by suppression of oxidative injury and inflammation. Free Radic. Res. 44 (8), 925–935. 10.3109/10715762.2010.490837 20815773

[B7] Ekinci Akdemir F. N. Yildirim S. Kandemir F. M. Aksu E. H. Guler M. C. Kiziltunc Ozmen H. (2019). The antiapoptotic and antioxidant effects of eugenol against cisplatin-induced testicular damage in the experimental model. Andrologia 51 (9), e13353. 10.1111/and.13353 31243800

[B8] El-Kady A. M. Ahmad A. A. Hassan T. M. El-Deek H. E. M. Fouad S. S. Althagfan S. S. (2019). Eugenol, a potential schistosomicidal agent with anti-inflammatory and antifibrotic effects against Schistosoma mansoni, induced liver pathology. Infect. Drug Resist. 12, 709–719. 10.2147/IDR.S196544idr-12-709 30992676 PMC6445185

[B9] Fangjun L. Zhijia Y. (2018). Tumor suppressive roles of eugenol in human lung cancer cells. Thorac. Cancer 9 (1), 25–29. 10.1111/1759-7714.12508 29024500 PMC5754308

[B10] Feigin V. L. Roth G. A. Naghavi M. Parmar P. Krishnamurthi R. Chugh S. (2016). Global burden of stroke and risk factors in 188 countries, during 1990-2013: a systematic analysis for the Global Burden of Disease Study 2013. Lancet Neurol. 15 (9), 913–924. 10.1016/S1474-4422(16)30073-4 27291521

[B11] Feng J. Chen X. Guan B. Li C. Qiu J. Shen J. (2018). Inhibition of Peroxynitrite-induced mitophagy activation attenuates cerebral ischemia-reperfusion injury. Mol. Neurobiol. 55 (8), 6369–6386. 10.1007/s12035-017-0859-x 29307080

[B12] Forouzanfar M. H. Alexander L. Anderson H. R. Bachman V. F. Biryukov S. Brauer M. (2015). Global, regional, and national comparative risk assessment of 79 behavioural, environmental and occupational, and metabolic risks or clusters of risks in 188 countries 1990-2013: a systematic analysis for the global burden of disease study 2013. Lancet 386 (10010), 2287–2323. 10.1016/S0140-6736(15)00128-2 26364544 PMC4685753

[B13] Fu X. Jin L. Han L. Yuan Y. Mu Q. Wang H. (2019). miR-129-5p inhibits adipogenesis through autophagy and may be a potential biomarker for obesity. Int. J. Endocrinol. 2019, 5069578. 10.1155/2019/5069578 31781210 PMC6875017

[B14] Goodall M. L. Fitzwalter B. E. Zahedi S. Wu M. Rodriguez D. Mulcahy-Levy J. M. (2016). The autophagy machinery controls cell death switching between apoptosis and necroptosis. Dev. Cell 37 (4), 337–349. 10.1016/j.devcel.2016.04.018 27219062 PMC4886731

[B15] Hansen T. E. Johansen T. (2011). Following autophagy step by step. BMC Biol. 9, 39. 10.1186/1741-7007-9-39 21635796 PMC3107173

[B16] Hou J. Rao M. Zheng W. Fan J. Law B. Y. K. (2019). Advances on cell autophagy and its potential regulatory factors in renal ischemia-reperfusion injury. DNA Cell Biol. 38 (9), 895–904. 10.1089/dna.2019.4767 31347925

[B17] Itakura E. Mizushima N. (2009). Atg14 and UVRAG: mutually exclusive subunits of mammalian Beclin 1-PI3K complexes. Autophagy 5 (4), 534–536. 10.4161/auto.5.4.8062 19223761

[B18] Jo H. K. Kim G. W. Jeong K. J. Kim D. Y. Chung S. H. (2014). Eugenol ameliorates hepatic steatosis and fibrosis by down-regulating SREBP1 gene expression via AMPK-mTOR-p70S6K signaling pathway. Biol. Pharm. Bull. 37 (8), 1341–1351. 10.1248/bpb.b14-00281 25087956

[B19] Kim J. Kundu M. Viollet B. Guan K. L. (2011). AMPK and mTOR regulate autophagy through direct phosphorylation of Ulk1. Nat. Cell Biol. 13 (2), 132–141. 10.1038/ncb2152ncb2152 21258367 PMC3987946

[B20] Kim J. H. Hong S. K. Wu P. K. Richards A. L. Jackson W. T. Park J. I. (2014). Raf/MEK/ERK can regulate cellular levels of LC3B and SQSTM1/p62 at expression levels. Exp. Cell Res. 327 (2), 340–352. 10.1016/j.yexcr.2014.08.001S0014-4827(14)00329-2 25128814 PMC4164593

[B21] Kumar A. Dhawan A. Kadam A. Shinde A. (2018). Autophagy and mitochondria: targets in neurodegenerative disorders. CNS Neurol. Disord. Drug Targets 17 (9), 696–705. 10.2174/1871527317666180816100203CNSNDDT-EPUB-92410 30113005

[B22] Lekoubou A. Awoumou J. J. Kengne A. P. (2017). Incidence of seizure in stroke patients treated with recombinant tissue plasminogen activator: a systematic review and meta-analysis. Int. J. Stroke 12 (9), 923–931. 10.1177/1747493017729239 28872451

[B23] Lin T. K. Chen S. D. Chuang Y. C. Lin H. Y. Huang C. R. Chuang J. H. (2014). Resveratrol partially prevents rotenone-induced neurotoxicity in dopaminergic SH-SY5Y cells through induction of heme oxygenase-1 dependent autophagy. Int. J. Mol. Sci. 15 (1), 1625–1646. 10.3390/ijms15011625ijms15011625 24451142 PMC3907890

[B24] Lo E. H. Dalkara T. Moskowitz M. A. (2003). Mechanisms, challenges and opportunities in stroke. Nat. Rev. Neurosci. 4 (5), 399–415. 10.1038/nrn1106nrn1106 12728267

[B25] Meley D. Bauvy C. Houben-Weerts J. H. Dubbelhuis P. F. Helmond M. T. Codogno P. (2006). AMP-activated protein kinase and the regulation of autophagic proteolysis. J. Biol. Chem. 281 (46), 34870–34879. 10.1074/jbc.M605488200 16990266

[B26] Mizushima N. Komatsu M. (2011). Autophagy: renovation of cells and tissues. Cell 147 (4), 728–741. 10.1016/j.cell.2011.10.026S0092-8674(11)01276-1 22078875

[B27] Nazio F. Strappazzon F. Antonioli M. Bielli P. Cianfanelli V. Bordi M. (2013). mTOR inhibits autophagy by controlling ULK1 ubiquitylation, self-association and function through AMBRA1 and TRAF6. Nat. Cell Biol. 15 (4), 406–416. 10.1038/ncb2708ncb2708 23524951

[B28] Nitatori T. Sato N. Waguri S. Karasawa Y. Araki H. Shibanai K. (1995). Delayed neuronal death in the CA1 pyramidal cell layer of the gerbil hippocampus following transient ischemia is apoptosis. J. Neurosci. 15 (2), 1001–1011. 10.1523/JNEUROSCI.15-02-01001.1995 7869078 PMC6577848

[B29] Roger V. L. Go A. S. Lloyd-Jones D. M. Benjamin E. J. Berry J. D. Borden W. B. (2012). Executive summary: heart disease and stroke statistics–2012 update: a report from the American Heart Association. Circulation 125 (1), 188–197. 10.1161/CIR.0b013e3182456d46125/1/188 22215894

[B30] Schaaf M. B. Keulers T. G. Vooijs M. A. Rouschop K. M. (2016). LC3/GABARAP family proteins: autophagy-(un)related functions. FASEB J. 30 (12), 3961–3978. 10.1096/fj.201600698R 27601442

[B31] Shen P. Hou S. Zhu M. Zhao M. Ouyang Y. Feng J. (2017). Cortical spreading depression preconditioning mediates neuroprotection against ischemic stroke by inducing AMP-activated protein kinase-dependent autophagy in a rat cerebral ischemic/reperfusion injury model. J. Neurochem. 140 (5), 799–813. 10.1111/jnc.13922 27987215

[B32] Sica V. Galluzzi L. Bravo-San Pedro J. M. Izzo V. Maiuri M. C. Kroemer G. (2015). Organelle-specific initiation of autophagy. Mol. Cell 59 (4), 522–539. 10.1016/j.molcel.2015.07.021S1097-2765(15)00579-1 26295960

[B33] Sun Q. Fan W. Chen K. Ding X. Chen S. Zhong Q. (2008). Identification of Barkor as a mammalian autophagy-specific factor for Beclin 1 and class III phosphatidylinositol 3-kinase. Proc. Natl. Acad. Sci. U. S. A. 105 (49), 19211–19216. 10.1073/pnas.08104521050810452105 19050071 PMC2592986

[B34] Sun J. Mu Y. Jiang Y. Song R. Yi J. Zhou J. (2018a). Inhibition of p70 S6 kinase activity by A77 1726 induces autophagy and enhances the degradation of superoxide dismutase 1 (SOD1) protein aggregates. Cell Death Dis. 9 (3), 407. 10.1038/s41419-018-0441-0 29540819 PMC5851998

[B35] Sun Y. Zhang T. Zhang Y. Li J. Jin L. Shi N. (2018b). Ischemic postconditioning alleviates cerebral ischemia-reperfusion injury through activating autophagy during early reperfusion in rats. Neurochem. Res. 43 (9), 1826–1840. 10.1007/s11064-018-2599-3 30046966 PMC6096887

[B36] Sun C. M. Enkhjargal B. Reis C. Zhou K. R. Xie Z. Y. Wu L. Y. (2019). Osteopontin attenuates early brain injury through regulating autophagy-apoptosis interaction after subarachnoid hemorrhage in rats. CNS Neurosci. Ther. 25 (10), 1162–1172. 10.1111/cns.13199 31436915 PMC6776743

[B37] Wang P. Shao B. Z. Deng Z. Chen S. Yue Z. Miao C. Y. (2018). Autophagy in ischemic stroke. Prog. Neurobiol. 163-164, 98–117. 10.1016/j.pneurobio.2018.01.001 29331396

[B38] Weichhart T. (2012). Mammalian target of rapamycin: a signaling kinase for every aspect of cellular life. Methods Mol. Biol. 821, 1–14. 10.1007/978-1-61779-430-8_1 22125056

[B39] Won M. H. Lee J. C. Kim Y. H. Song D. K. Suh H. W. Oh Y. S. (1998). Postischemic hypothermia induced by eugenol protects hippocampal neurons from global ischemia in gerbils. Neurosci. Lett. 254 (2), 101–104. 10.1016/s0304-3940(98)00664-8 9779930

[B40] Wu M. Zhang H. Kai J. Zhu F. Dong J. Xu Z. (2018). Rapamycin prevents cerebral stroke by modulating apoptosis and autophagy in penumbra in rats. Ann. Clin. Transl. Neurol. 5 (2), 138–146. 10.1002/acn3.507ACN3507 29468175 PMC5817831

[B41] Yao X. Yao R. Huang F. Yi J. (2019). LncRNA SNHG12 as a potent autophagy inducer exerts neuroprotective effects against cerebral ischemia/reperfusion injury. Biochem. Biophys. Res. Commun. 514 (2), 490–496. 10.1016/j.bbrc.2019.04.158 31056262

[B42] Zhang X. Yan H. Yuan Y. Gao J. Shen Z. Cheng Y. (2013). Cerebral ischemia-reperfusion-induced autophagy protects against neuronal injury by mitochondrial clearance. Autophagy 9 (9), 1321–1333. 10.4161/auto.25132 23800795

[B43] Zhang D. M. Zhang T. Wang M. M. Wang X. X. Qin Y. Y. Wu J. (2019a). TIGAR alleviates ischemia/reperfusion-induced autophagy and ischemic brain injury. Free Radic. Biol. Med. 137, 13–23. 10.1016/j.freeradbiomed.2019.04.002 30978385

[B44] Zhang Y. Jin X. F. Zhou X. H. Dong X. H. Yu W. T. Gao W. J. (2019b). the role of astragaloside IV against cerebral ischemia/reperfusion injury: suppression of apoptosis via promotion of P62-LC3-autophagy. Molecules 24 (9), 1838. 10.3390/molecules24091838molecules24091838 PMC6539971 31086091

[B45] Zhao Y. Xue Y. Liu Z. Ren S. Guan X. Li M. (2019). Role of the Janus kinase 2/signal transducers and activators of transcription 3 pathway in the protective effect of remote ischemia preconditioning against cerebral ischemia-reperfusion injury in rats. Neuroreport 30 (9), 664–670. 10.1097/WNR.0000000000001257 30969244 PMC6530975

[B46] Zhou X. Y. Luo Y. Zhu Y. M. Liu Z. H. Kent T. A. Rong J. G. (2017). Inhibition of autophagy blocks cathepsins-tBid-mitochondrial apoptotic signaling pathway via stabilization of lysosomal membrane in ischemic astrocytes. Cell Death Dis. 8 (2), e2618. 10.1038/cddis.2017.34cddis201734 28206988 PMC5386481

