# Supplementary figures and images for: Eugenol Attenuates Cerebral Ischemia-Reperfusion Injury by Enhancing Autophagy via AMPK-mTOR-P70S6K Pathway
Source: Front Pharmacol. 2020 Feb 21;11:84. doi: 10.3389/fphar.2020.00084 (PMC7047211; doi:10.3389/fphar.2020.00084)

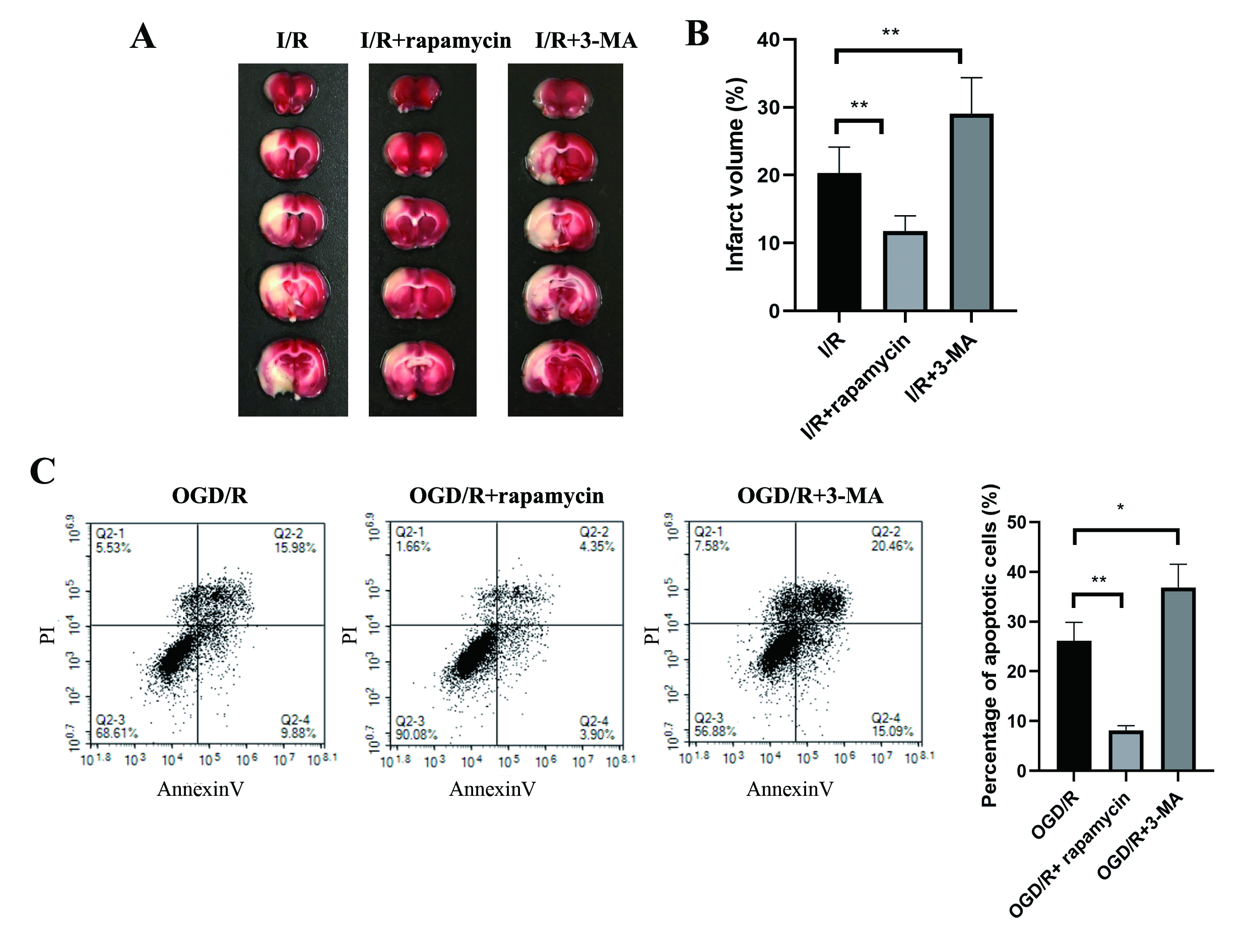

Supplement: Figure S1 — Cerebral I/R injury was attenuated by autophagy. Cerebral I/R injury was induced by MCAO in mice. Autophagy was promoted by pretreatment with rapamycin, or inhibited by pretreatment with 3-MA. (A) After reperfusion for 24 h, TTC staining assay was performed to evaluate infarct volume. (B) The infarct volume of different groups was calculated and shown. HT22 cells were pretreated with rapamycin (1 μM) or 3-MA (5 mM) for 1 h, and then subjected to OGD/R. (C) The apoptosis of HT22 cells was determined by AnnexinV/PI staining and the percentage of apoptotic cells was calculated. Each experimental datum was presented as mean±standard deviation (n = 6 for A&B, n = 3 for C). *P < 0.05, **P < 0.01 versus the indicated group. [file Image_1.jpeg]

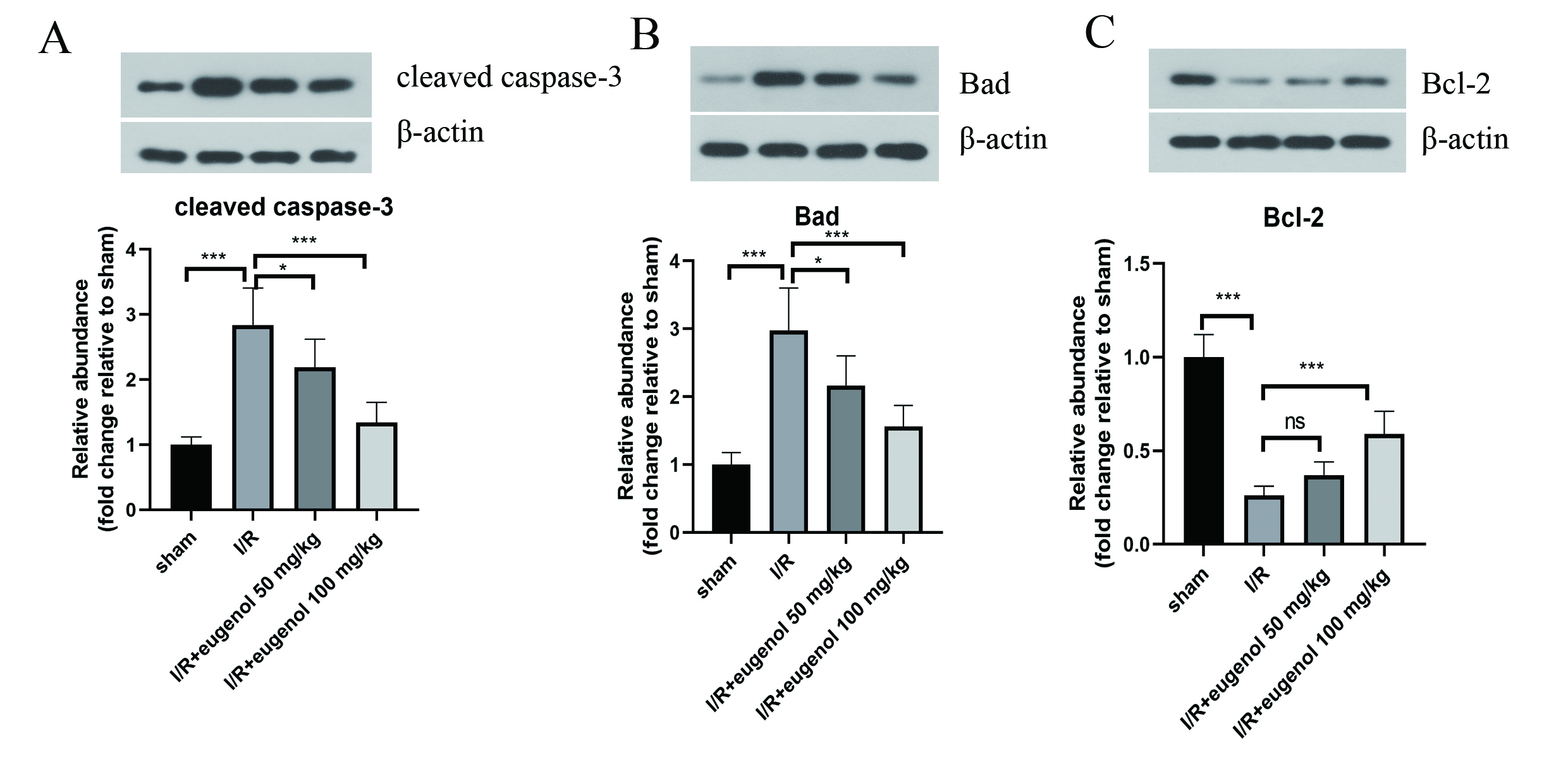

Supplement: Figure S2 — Effect of eugenol on the expression of apoptotic markers. (A–C) The protein levels of cleaved caspase-3, Bad, and Bcl-2 in the brain tissues were detected by Western blotting assay. Each experimental datum was presented as mean±standard deviation (n = 6). *P < 0.05, ***P < 0.001 versus the indicated group. [file Image_2.jpeg]
